# Computational thinking and thinking about computing

**DOI:** 10.1098/rsta.2008.0118

**Published:** 2008-07-31

**Authors:** Jeannette M. Wing

**Affiliations:** Computer Science Department, Carnegie Mellon UniversityPittsburgh, PA 15213, USA

**Keywords:** computational thinking, abstraction, automation, computing, computable, intelligence

## Abstract

Computational thinking will influence everyone in every field of endeavour. This vision poses a new educational challenge for our society, especially for our children. In thinking about computing, we need to be attuned to the three drivers of our field: science, technology and society. Accelerating technological advances and monumental societal demands force us to revisit the most basic scientific questions of computing.

## 1. Computational thinking

Computational thinking is taking an approach to solving problems, designing systems and understanding human behaviour that draws on concepts fundamental to computing^1^ (Wing 2006).

Computational thinking is a kind of analytical thinking. It shares with mathematical thinking in the general ways in which we might approach solving a problem. It shares with engineering thinking in the general ways in which we might approach designing and evaluating a large, complex system that operates within the constraints of the real world. It shares with scientific thinking in the general ways in which we might approach understanding computability, intelligence, the mind and human behaviour.

### (a) Computing: abstraction and automation

The essence of computational thinking is *abstraction*. In computing, we abstract notions beyond the physical dimensions of time and space. Our abstractions are extremely general because they are symbolic, where numeric abstractions are just a special case.

In two ways, our abstractions tend to be richer and more complex than those in the mathematical and physical sciences. First, our abstractions do not necessarily enjoy the clean, elegant or easily definable algebraic properties of mathematical abstractions, such as real numbers or sets, of the physical world. For example, a stack of elements is a common abstract data type used in computing. We would not think ‘to add’ two stacks as we would two integers. An algorithm is an abstraction of a step-by-step procedure for taking input and producing some desired output. What does it mean ‘to interleave’ two algorithms, perhaps for efficient parallel processing? A programming language is an abstraction of a set of strings each of which when interpreted effects some computation. What does it mean ‘to combine’ two programming languages? These kinds of combinators are themselves abstractions that take careful thought, perhaps an entire research agenda, to define. Second, because our abstractions are ultimately implemented to work within the constraints of the physical world, we have to worry about edge cases and failure cases. What happens when the disk is full or the server is not responding? What happens when a program encounters at run-time an error that should have been caught at compile time? How do we get a robot to move down a hallway without bumping into people?

In working with rich abstractions, defining the ‘right’ abstraction is critical. The abstraction process—deciding what details we need to highlight and what details we can ignore—underlies computational thinking.

The abstraction process introduces layers. In computing, we work simultaneously with at least two, usually more, layers of abstraction: the layer of interest and the layer below; or the layer of interest and the layer above. Well-defined interfaces between layers enable us to build large, complex systems. Given the application programming interface (API) of a software component, a user need not know the details of the component's implementation to know how to interact with it, and an implementer need not know who all the component's potential users might be in order to implement it correctly. The layered architecture of the Internet, in particular the ‘thin waist’ Internet protocol layer, supports both the incorporation of new computing devices and networking technology at the bottom and the addition of new, unforeseen applications at the top.

In working with layers of abstraction, we necessarily keep in mind the relationship between each pair of layers, be it defined via an abstraction function, a simulation relation, a transformation or a more general kind of mapping. We use these mappings in showing the observable equivalence between an abstract state machine and one of its possible refinements, in proving the correctness of an implementation with respect to a specification and in compiling a program written in a high-level language to more efficient machine code.

And so the nuts and bolts in computational thinking are defining abstractions, working with multiple layers of abstraction and understanding the relationships among the different layers. Abstractions are the ‘mental’ tools of computing.

The power of our ‘mental’ tools is amplified by the power of our ‘metal’ tools. Computing is the automation of our abstractions. We operate by mechanizing our abstractions, abstraction layers and their relationships. Mechanization is possible due to our precise and exacting notations and models. Automation implies the need for some kind of computer to interpret the abstractions. The most obvious kind of computer is a machine, i.e. a physical^2^ device with processing, storage and communication capabilities. Yes, a computer could be a machine, but more subtly it could be a human. Humans process information; humans compute. In other words, computational thinking does not require a machine. Moreover, when we consider the combination of a human and a machine as a computer, we can exploit the combined processing power of a human with that of a machine. For example, humans are still better than machines at parsing and interpreting images; on the other hand, machines are much better at executing certain kinds of instructions far more quickly than humans and processing datasets far larger than a human can handle.

Operationally, computing is concerned with answering ‘How would I get a computer to solve this problem?’ where the computer could be a machine, a human, the combination of a machine and a human, or recursively, the combination (e.g. a network) of such computers. Implicit in answering this question is our identifying appropriate abstractions and choosing the appropriate kind of computer for the task. Unfortunately, it is all too easy to answer this question by not thinking very hard about defining the right abstraction and then choosing a machine with lots of horsepower to solve the problem using brute force. Computational thinking can offer more than this simple use of mechanical computers.

### (b) Computational thinking everywhere

‘Computational thinking is influencing research in nearly all disciplines, both in the sciences and the humanities’ (Bundy 2007). Evidence of computational thinking's influence on other fields abounds: computational thinking is transforming statistics, where with machine learning the automation of Bayesian methods and the use of probabilistic graphical models make it possible to identify patterns and anomalies in voluminous datasets as diverse as astronomical maps, functional magnetic resonance imaging scans, credit card purchases and grocery store receipts (e.g. Machine Learning Department 2008). Computational thinking is transforming biology, first with the shotgun sequencing algorithm accelerating our ability to sequence the human genome, and now with our abstractions representing dynamic processes found in nature, from the cell cycle to protein folding (e.g. Fisher & Henzinger 2007). Computational thinking is transforming economics, spawning a new field of computational microeconomics, with applications such as advertisement placement, online auctions, reputation services and even finding optimal donors for *n*-way kidney exchange (Abraham *et al*. 2007).

In other fields, computational thinking is still at the stage of *simple* computational thinking: spending days' worth of machine cycles to solve problems. Many sciences and engineering disciplines rely on enormous computer simulations of mathematical models of physical processes found in nature. Aerospace relies on being able to simulate an entire aircraft or space mission. The geosciences dare to want to simulate the Earth, from its inner core to its surface to the Sun. In the humanities and the arts, digital libraries of books, collections and artefacts create opportunities through computational methods such as data mining and data federation to discover new trends, patterns and links in our understanding and appreciation of humankind.

Looking to the future, *deeper* computational thinking—through the choice of cleverer or more sophisticated abstractions—may enable scientists and engineers to model and analyse their systems on a scale orders of magnitude greater than they are able to handle today. Through the use of abstraction layers, e.g. hierarchical decomposition, we look forward to when we can: model systems at multiple time scales and at multiple resolutions of the three space dimensions; model the interactions of these many complex systems to identify conditions for tipping points and emergent behaviour; increase the number of parameters and sets of initial conditions in these models; play these models backwards and forwards in time; and validate these models against ground truth.

Deeper computational thinking will help us not only to model more and more complex systems, but also to analyse the massive amounts of data we collect and generate. Through deployment of distributed sensor nets, routine use of monitoring and surveillance systems, the prevalence of digital cameras on mobile (cell) phones, digitizing the world's information, running simulations of models of complex systems, and so on, we will be collecting and generating more and more data to analyse. It will be through computational thinking—abstractions for representing and processing the data—that we will be able to extract the knowledge buried within or spread throughout the data. There is an open feedback loop: this knowledge, piquing our curiosity, will lead us to ask new questions that require collection of more data; and this knowledge will help us to fine-tune our simulation models, thereby generating even more data.

*Vision no. 1*. I envision that computational thinking will be instrumental to new discovery and innovation in all fields of endeavour.

### (c) Computational thinking for everyone

If computational thinking will be used everywhere, then it will touch everyone directly or indirectly. This raises an educational challenge. If computational thinking is added to the repertoire of thinking abilities, then how and when should people learn this kind of thinking and how and when should we teach it? Let us assume that the trend of using computational thinking in research in all fields is already happening, thereby already influencing the training of graduate students. Let us further assume that universities have already begun to incorporate computational thinking in their undergraduate curricula, thereby recognizing how the next generation will have to be able to think in order to succeed in modern society. Thus, let us focus this question at the elementary through high school levels of education. In fact, if we wanted to ensure a common and solid basis of understanding and applying computational thinking for all, then this learning should best be done in the early years of childhood.

I pose the following as a challenge to the computer science, learning sciences and education communities.

*Challenge no. 1*. What are effective ways of learning (teaching) computational thinking by (to) children?

This question raises even more fundamental questions:

*What are the elemental concepts of computational thinking?* Educators in computing have answered and continue to answer this question by creating courses, typically for first-year undergraduates, that focus on the principles of computing rather than just on computer programming skills. As the field of computing continues to mature, it is worth revisiting this question again, with a specific focus on earlier years.

Moreover, it is worth revisiting this question in collaboration with scholars in learning science and education. For example, what, if any, computational thinking concepts are as innate to human cognition as is the mathematical concept of numbers? Human vision is parallel processing. What tasks do we most naturally do or learn to do in parallel versus sequentially? Children experience notions of infinity and recursion through mathematics and language; naming and teaching these fundamental concepts early on in formal learning settings would provide powerful building blocks for computational thinking.

*What would be an effective ordering of concepts in teaching children as their learning ability progresses over the years?* By analogy, we teach numbers to children in kindergarten (when they are 5 years old), algebra in junior high (12 years old) and calculus in senior high (18 years old). There may be many possible ways to structure the progression of computational thinking concepts; which is the most effective for which kind of learner?

*How best should we integrate the tool with teaching the concepts?* Here and henceforth, let ‘the tool’ mean the computing machine (a particular ‘metal’ tool of §1*a*). Our field of computing is in a unique situation since not only are there computational concepts to teach but also there is a tool to teach. This tool provides some challenges and opportunities.

One challenge is that we do not want the tool to get in the way of understanding the concepts. We also do not want people just to be able to use the tool but not have learned the concepts (a case in point: using a calculator versus understanding arithmetic). Worse, we do not want people to come away thinking they understand the concepts because they are adept at using the tool. A second challenge is that we want to track the learning of how to use the tool with the order of learning of concepts. At what point do we introduce each of the powerful capabilities of a computing machine? At what point do we expose children to the intricacies of how the machine works? These questions are analogous to choosing the right abstraction where now the criteria are defined by learning ability.

One opportunity is that we can use the tool to reinforce the concepts we teach. Computing is cool: it is all about making abstractions come alive! Through effective visualization and animation, even at early grades we can viscerally show the difference between a polynomial-time algorithm and an exponential-time one or show that a tree is a special kind of graph; in later grades through acquiring programming skills, students can automate their own abstractions. Indeed, this tool can be useful for reinforcing not just computational thinking concepts but also concepts in other fields. A second opportunity is that most children today are facile with the mechanics of using the tool and are not afraid to explore and play with it. We can take advantage of the routine exposure children have to computational devices at home and in school today.

Given this last observation, we should also explore informal as well as formal learning. Learning takes place in many ways and outside the classroom: children teach each other; learn from parents and family; learn at home, in museums and in libraries; and learn through hobbies, surfing the Web and life experiences.

*Vision no. 2*. I envision that computational thinking will be an integral part of childhood education.

*Caveat*. There are many cultural, economic, political and social barriers in realizing this vision, especially in countries where the education system is not centrally controlled. Striving towards this vision could still yield worthwhile benefits.

## 2. Thinking about computing

The field of computing is driven by scientific questions, technological innovation and societal demands. I remind us of this point for two reasons. First, in our field, we are often so swept up by our technological advances or societal expectations that we forget that there are deep scientific questions that underlie our field. Second, for others outside of computing, it is important to explain that the weight of each, and moreover the combination of our three drivers—science, technology and society—make our field unique, indeed distinctive from other sciences, mathematics and engineering. Why not celebrate this distinction?

**Figure 1:**
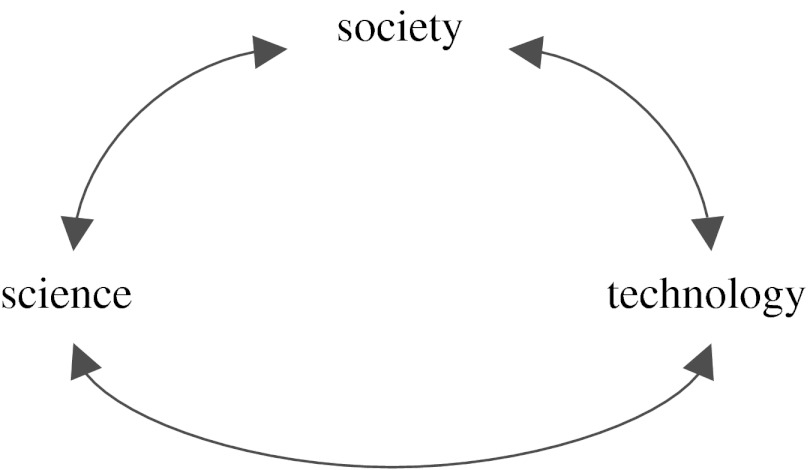
Three drivers of computing: science, technology and society.

Moreover, as shown by the bidirectional arrows in figure 1, there is wonderful interplay—push and pull—among these three drivers: in the usual loop, scientific discovery feeds technological innovation, which feeds new societal applications; in the reverse direction, new technology inspires new creative societal uses, which may demand new scientific discovery. An example of how society demands new science: the spread of our own computing and communications machinery, from mega-data centres of tens of thousands of servers to billions of mobile phones, requires new advances in science to use energy more efficiently. An example of how society demands new technology: the desire for higher fidelity and more realistic virtual environments is straining our network capability for real-time simultaneous transmission of multiple multimedia (audio, video and text) data streams. Another example of technology pull is how a fundamental social desire to express one's identity and connect with likeminded others led to the unanticipated and rapid rise of social networks, such as Facebook, MySpace and YouTube, which in turn added a new industry to our economy.

Our field most naturally anticipates technology trends and embraces the demands and expectations of society, so let us start with the technology drivers, then societal, and then scientific.

### (a) Technology drivers

Beginning at the computing substrate level, we are predicting the end of Moore's law within the next 10–15 years (Engadget 2006). The immediate consequence for silicon-based technology is the production of multi-core architecture machines; the challenge is understanding how to program them to use their parallel processing capability effectively.

Beyond silicon, we look at nanocomputing, biocomputing and even quantum computing. In some sense they have already arrived. Nano is here: IBM (2006) announced that its researchers had built the first complete integrated circuit around a single carbon nanotube molecule. Bio is here: Adleman (1994) solved the seven-point Hamiltonian path problem with DNA computing; Benenson *et al*. (2004) described in *Nature* the construction of a DNA computer. We are now building molecular machines. Quantum is coming? The Swiss (Messmer 2007) use quantum cryptography to secure ballots in their elections. The design of nanocomputers and bio-inspired computers must already take into consideration quantum effects (e.g. Heller *et al*. 2005).

At the device level, Strukov *et al*. (2008) announced that they can create a memristor (Chua 1971), the missing fourth element along with the resistor, capacitor and inductor. At larger scales, we see a growing use of mobile phones, radio frequency identification tags, sensors, actuators and robots. Our automobiles are laced with embedded computers: a BMW is ‘now actually a network of computers’ (Economist 2007).

In terms of data, we are drowning in data (cf. §1*b*). Sensors are everywhere, storage is cheap and we are in a constant state of information overload.

In terms of communication, Web 3.0 or the Semantic Web is an active area of research. We will see more sophisticated virtual worlds; Second Life is today's Mosaic. The scientists and engineers of tomorrow will conduct their work through virtual organizations, facilitating international collaboration.

In terms of far-reaching technological machines, people aspire to build machines that model the human brain. The IBM and EPFL's Blue Brain Project (2005) aims to create a biologically accurate, functional model of the brain. The start-up company Numenta (2005) is building a software platform for intelligent computing modelled after the human neocortex.

These are just a few technology trends of today; it will be interesting to read this section of this paper in 10 years to see where we were and how far we will have gone.

### (b) Societal drivers

The success of our information technology, including computers and communications, has raised society's expectations of us. People now demand availability, 24 hours per day, every day, 100 per cent reliability, 100 per cent connectivity, instantaneous response, the ability to store anything and everything forever, and the ability for anyone to access anything from anywhere at any time.

The classes of users of our technology are not limited to scientists and engineers. Rather, our users are young and old, able and disabled, rich and poor, literate and illiterate.

Our technology must also support a range in the number of users: from individual to groups to populations to the global society. Individuals want highly personalized devices and services; search companies realize this desire by tracking our queries and personalizing the advertisements we see. Cliques of friends lead to larger acquaintance networks such as LinkedIn or social networks such as Facebook. Different populations may use information and networking technology to preserve their cultural heritage.

The Internet and the World Wide Web together is a great equalizer. On the other hand, there remain scientific and technical challenges regarding accountability, anonymity, identity management and privacy.

*Challenge no. 2*. How do we make our technology and the wealth of our applications accessible to all? How do we balance openness with privacy?

### (c) Science drivers

Wing (2008) presented five ‘deep questions in computing’, as a way to remind ourselves that there are scientific challenges that underlie our individual research pursuits or innovations in technology. I repeat this set (i.e. no ordering implied) of questions for the sake of completeness in this paper. This set is meant to be a starting point, with new questions added by the entire community.

- Does P equal NP?
- What is computable?
- What is intelligence?
- What is information?
- (How) can we build complex systems simply?

*Closing question*. Given (i) the philosophy of §1*a*, which says that computational thinking is informed by our desire to automate abstractions, where the computer can be human and/or machine, and (ii) the technological trends outlined in §2*a*, which test the adequacy of Shannon's information theory and the Turing machine as the fundamental model of computation, we might even ask the most basic question of all: *what is a computer?*

## Footnotes

By ‘computing’ I mean very broadly the field encompassing computer science, computer engineering, communications, information science and information technology.

The obvious physical devices are the mechanical or electrical of today. I also mean to include the physical devices of tomorrow, e.g. nano and quantum computers; and even the biological devices of tomorrow, e.g. organic, DNA and molecular computers, as well (cf. §2*a*).
